# Quantitative trait loci for yellow rust resistance in spring wheat doubled haploid populations developed from the German Federal ex situ genebank genetic resources

**DOI:** 10.1002/tpg2.20142

**Published:** 2021-09-09

**Authors:** Ibrahim S. Draz, Albrecht Serfling, Quddoos H. Muqaddasi, Marion S. Röder

**Affiliations:** ^1^ Wheat Disease Research Dep., Plant Pathology Research Institute Agricultural Research Center 9 Gamaa Street Giza 12619 Egypt; ^2^ Julius Kühn Institute—Federal Research Centre for Cultivated Plants Institute for Resistance Research and Stress Tolerance Erwin Baur Straße 27 Quedlinburg 06484 Germany; ^3^ Leibniz Institute of Plant Genetics and Crop Plant Research Corrensstraße 3, 06466, Stadt Seeland OT Gatersleben Germany; ^4^ Present address: European Wheat Breeding Center BASF Agricultural Solutions GmbH Am Schwabeplan 8, 06466, Stadt Seeland OT Gatersleben Germany

## Abstract

Novel resistance sources to the pathogen *Puccinia striiformis* f. sp. *tritici*, which causes yellow rust (stripe rust), a widespread devastating foliar disease in wheat (*Triticum aestivum* L.), are in demand. Here, we tested two doubled haploid (DH) spring wheat populations derived from the genetic resources for resistance to yellow rust in field trials in Germany and Egypt. Additionally, we performed tests for all‐stage resistance (seedling resistance). We performed linkage mapping based on 15k Infinium SNP chip genotyping data that resulted in 3,567 and 3,457 polymorphic markers for DH Population 1 (103 genotypes) and DH Population 2 (148 genotypes), respectively. In DH Population 1, we identified a major and consistent quantitative trait locus (QTL) on chromosome 1B that explained up to 28 and 39% of the phenotypic variation in the field and seedling tests, respectively. The favorable allele was contributed by the line ‘TRI‐5645’, a landrace from Iran, and is most probably the yellow rust resistance (*Yr*) gene *Yr10*. In DH Population 2, the favorable allele of a major QTL on chromosome 6B was contributed by the line ‘TRI‐5310’, representing the variety ‘Eureke’ from France. This QTL was mainly effective in the German environments and explained up to 36% of the phenotypic variation. In Egypt, however, only a moderate resistance QTL was identified in the field tests and no resistance QTL was observed in the seedling tests. Our results demonstrate the usefulness of genetic resources to identify novel sources of resistance to yellow rust, including the “Warrior” race *PstS10*.

## Core Ideas

-  Major QTLs for yellow rust resistance were discovered in genetic resources of wheat.
-  Resistance patterns were environment‐specific and included resistance to the race “Warrior”.
-  Physical regions on chromosomes 1B and 6B were evaluated for candidate genes.
-  Resistance on chromosome 1B is most probably conferred by the gene *Yr10*.

**Abbreviations**
APR: adult plant resistance
BBCH: Biologische Bundesanstalt für Land‐ und Forstwirtschaft, Bundessortenamt und Chemische Industrie
DH: doubled haploid
*Pst*
: *Puccinia striiformis* Westend. f. sp. *tritici*
QTL: quantitative trait locus
SNP: single nucleotide polymorphisms
*Yr*
: yellow rust resistance (gene)

## 1 INTRODUCTION

Yellow rust (stripe rust), caused by *Puccinia striiformis* Westend. f. sp. *tritici* Erikss. (*Pst*), is one of the most destructive foliar diseases that threatens the food security particularly in temperate, humid, and cooler wheat (*Triticum aestivum* L.)‐growing regions of the world (Chen, 2005, 2020; Kumar et al., 2020). Depending upon the cultivar's susceptibility, time of initial infection, disease period, and development rate, the disease causes significant yield losses that may range from 10 to 70% (Chen, 2005). Nevertheless, following initial infection at an early growth stage, it may also result in up to 100% of yield losses. Up to 88% of the world's wheat cultivars have become susceptible since 1960, with annual losses amounting to 5.47 million tons (Beddow et al., 2015). Extensive studies on *Pst* races were conducted by Gassner and Straib (1933) in Germany in the 1930s. Up to 2010, yellow rust occurred infrequently and had only a minor effect on varieties of small‐grain cereals in continental Europe, mainly by deploying a few long‐term effective major resistance genes (de Vallavieille‐Pope et al., 2012). In 2011, however, virulent yellow rust races, termed “Warrior” and “Warrior (–)”, were found on both wheat and triticale. These races simultaneously emerged in several European countries and have rapidly spread over most of the continent, resulting in a breakdown of race‐specific *Yr* resistance genes carried by many cultivars (Hovmøller et al., 2016). According to monitoring performed by the federal Julius Kühn Institute in Germany (Hovmøller et al., 2016), the *Pst* race Warrior dominated the yellow rust population in Germany in 2015 and 2016, and only a few yellow rust resistance (*Yr*) genes (i.e., *Yr5*, *Yr8*, *Yr10*, *Yr15*, and *Yr24*) are still effective.

In Egypt, the disease has affected most commercial wheat varieties, and a major epiphytotic event has been recorded once every decade since the 1960s. This has resulted in grain yield losses that ranged from 14 to 26% in the Nile Delta region and a 10% loss countrywide (El‐Daoudi et al., 1996). In recent years, the pathotypic evolution of *Pst* has increased dramatically; as a result, many wheat cultivars have lost their resistance and have eventually become susceptible. For example, grain yield losses of up to 23.12% were reported in the cultivar Giza‐160 (Draz, 2019b; Draz et al., 2018). Coupled with crop management practices, deploying genetically resistant varieties is deemed to be economical, sustainable, and environmental‐friendly way to control the diseases (Chen, 2013; Pink, 2002; Yang et al., 2019).

Genetic resistance to yellow rust in wheat can be broadly categorized as (a) race‐specific seedling resistance (all‐stage resistance), which is detected at the seedling stage but is also expressed at all stages of plant growth; (b) race‐specific adult plant resistance (APR), which is expressed at later stages of plant growth; and (c) race non‐specific APR that is also known as slow‐rusting or partial resistance (Chen, 2013; Das et al., 1992). To date, more than 80 *Yr* genes have been named in wheat (Feng et al., 2018; Long et al., 2019; McIntosh et al., 2017; Nsabiyera et al., 2018); of these, 67 *Yr* genes have been temporarily designated as seedling resistance and APR (Wang & Chen, 2017). Most of these *Yr* genes are race‐specific and liable to be overcome by new virulent races. For instance, *Yr11*–*Yr14*, *Yr16*, *Yr39*, and *Yr49* confer race‐specific APR, whereas *Yr2*, *Yr4*–*Yr10*, *Yr15*, *Yr17*, *Yr19*, *Yr25* to *Yr28*, *Yr35* to *Yr38*, *Yr40*, *Yr42*, *Yr53*, *Yr61*, *Yr65*, and *Yr69* confer seedling resistance (Chen, 2005; Zheng et al., 2017). *Yr18*, *Yr29*, and *Yr46*, however, confer non‐ race‐specific APR. Nevertheless, most *Yr* genes are unique, have different chromosomal locations, respond differently to pathogen races, and originate from different wheat genotypes or wild species. As stated elsewhere, the most effective strategy to protect wheat from yellow rust is to deploy cultivars with all‐stage resistance and APR genes for durable resistance (Chen, 2005). Molecular markers linked to genes for resistance to yellow rust have been previously identified in biparental populations obtained by crossing resistant and susceptible wheat genotypes (William et al., 2003). Over the past 20 yr, ∼327 quantitative trait loci (QTLs) for resistance to yellow rust have been reported (McIntosh et al., 2017; Wang & Chen, 2017) via a wide range of markers, including diversity array technology, simple sequence repeats, and single nucleotide polymorphisms (SNPs) (Gebrewahid et al., 2020; Lan et al., 2017; Maccaferri et al., 2015; Rosewarne et al., 2013). The genetic locations of these QTLs are continually being refined through subsequent mapping studies. Because of their high throughput, efficiency, allele specificity, and high‐resolution capacity, the development of SNP markers—which are abundant, co‐dominant, and present throughout the wheat genome—has revolutionized wheat research (Allen et al., 2013; Chen et al., 1998; Gupta et al., 2008; Kumar et al., 2020; Long et al., 2019; Röder et al., 1998; Zhou et al., 2014). In recent studies, novel SNP‐based QTLs for yellow rust resistance have been detected in different types of wheat (Gebrewahid et al., 2020; Wu et al., 2018; Zheng et al., 2017).

**Core Ideas**

-  Major QTLs for yellow rust resistance were discovered in genetic resources of wheat.
-  Resistance patterns were environment‐specific and included resistance to the race “Warrior”.
-  Physical regions on chromosomes 1B and 6B were evaluated for candidate genes.
-  Resistance on chromosome 1B is most probably conferred by the gene *Yr10*.

The available host genetic resources for resistance to yellow rust are still limited and are liable to be overcome by the pathogen because of constant evolution (Chen, 2005; Roelfs, 1992). Despite the huge impact of the Warrior race on wheat grain production in Europe, little is known about the genetic control of the resistance to this novel yellow rust race, which is the basis of knowledge‐based resistance breeding. This warrants the need for new sources of resistance. We aimed to identify potentially novel QTLs for yellow rust resistance by using QTL mapping on two doubled haploid (DH) populations developed from the spring wheat accessions from the German Federal ex situ genebank by phenotypic testing in German and Egyptian environments. We detected novel large‐effect QTLs and used the genomic sequence to identify potential candidate genes. Since genetic resources hold a good promise for mining untapped disease resistance, these QTLs could be used in prebreeding programs to develop resistance in elite germplasm. In addition, these QTLs serve as a source for future map‐based cloning studies.

## 2 MATERIALS AND METHODS

### 2.1 Plant material

The two DH spring wheat populations have previously been described (Muqaddasi et al., 2019). The parental lines were genetic resources obtained from the genebank of the Leibniz Institute of Plant Genetics and Crop Plant Research in Gatersleben (https://www.ipk‐gatersleben.de/en/gbisipk‐gaterslebendegbis‐i/). For DH Population 1, consisting of 103 genotypes, the parental lines were ‘TRI‐11082’ (the former German Democratic Republic variety ‘Hatri’) and ‘TRI‐5645’ (a landrace from Iran), whereas the parental lines of DH Population 2, consisting of 148 genotypes, were ‘TRI‐10703’ (a landrace from Greece) and ‘TRI‐5310’ (the variety ‘Eureke’ from France). These populations were originally developed to assess and map anther extrusion in the field (Muqaddasi et al., 2019), where segregation for yellow rust infection was observed. Therefore, the current study was initiated to follow up on the genetic mapping of yellow rust resistance.

### 2.2 Field tests for resistance to yellow rust in Germany

A field test for resistance to yellow rust was conducted at the Julius Kühn Institute in Quedlinburg (51°46′05.7″N 11°8′54.9″E) in a randomized incomplete block design with two replications. Each plot consisted of two rows with a length of 1 m. The distance between the rows was 20 cm. Additional spreader rows of the susceptible variety ‘Triso’ (Deutsche Saatveredlung AG) were arranged regularly in every third plot. Per row, 25 seeds were sown in the middle of March. To ensure uniform infestation, the spreader stripes were artificially inoculated at growth stage according to Biologische Bundesanstalt für Land‐ und Forstwirtschaft, Bundessortenamt und Chemische Industrie (BBCH)‐33 [three nodes detectable, as described in Lancashire et al. (1991)]. A spore suspension of 10 mg uredospores in 100 ml Isopar M (ExxonMobil Chemical Company) was applied in a total amount of 10 ml suspension m^–^^2^ with a hand‐held spinning disc sprayer (ULVA+, Micron Sprayers). Spreaders were inoculated with the *PstS10* urediniospores of the Warrior race, kindly provided by Kerstin Flath (Julius Kühn Institute, Institute for Plant Protection in Field Crops and Grassland, Kleinmachnow, Germany). Because of the uniform infection level in plots, the average stripe rust severity was detected as the percentage of infected leaf area (Moll et al., 2010) 30, 37, and 43 d after inoculation. The area under the disease pressure curve was estimated for each population (Jeger & Viljanen‐Rollinson, 2001; Moll et al., 2010).

The field test was repeated in 2019 at Gatersleben, Germany (51°49′N 11°16′E, 112 m asl) in one replication without a specific design. Readings of infection percentages differed from the 2018 field test in Quedlinburg for practical reasons and were conducted 43, 51, and 56 d after inoculation, and area under the disease pressure curve values were determined. The area under the disease pressure curve values in both German trials were estimated manually according to the formula:

$$\left[\left(\frac{A+B}{2}\right)\times N\right]+\left[\left(\frac{C-B}{2}\right)\times M\right]$$

where *A* is the  disease score at the first evaluation, *B* is the disease score at the second evaluation, *C* is the disease score at the third evaluation, *N* is the number of  days between the first and second evaluations, and *M* is the number of  days between the second and third evaluations.

### 2.3 Seedling tests in Egypt

The two DH populations and their parents were tested for seedling resistance to yellow rust in the greenhouse of the Wheat Disease Research Department, Sakha Agricultural Research Station, Plant Pathology Research Institute, Agricultural Research Centre, Egypt, during the 2017–2018 and 2018–2019 growing seasons. The experiment was carried out in a completely randomized design with four replications. The tested material was grown in plastic pots (10 cm diameter) in clay soil; each pot received 10 kernels. Eight‐day‐old seedlings were inoculated with urediniospores of *Pst* isolates according to the method described by Simmonds and Rajaram (1988). The inoculum of *Pst* isolates represented a mixture of the prevailing races in Egypt during the respective growing season, including the aggressive *PstS2* race. The inoculated seedlings were misted with water and incubated in a dark dew chamber at 10 °C for 24 h under high relative humidity. After incubation, the inoculated seedlings were transferred to a greenhouse conditioned at 100% relative humidity and 13 ± 2 °C under a 16‐h photoperiod with a light intensity of 100 μmol m^−2^ s^−1^. Disease scoring was performed 16 to 18 days after inoculation as infection types on a 0 to 9 scale (Line & Qayoum, 1992; McNeal et al., 1971). The first seedling leaf was considered for the phenotypic trait, where infection types 0 to 6 refer to the resistant response and types 7 to 9 indicated susceptibility (Wan & Chen, 2014).

### 2.4 Field resistance tests in Egypt

The two DH populations and their parents were tested for APR to yellow rust at the Experimental Farm of Sakha Agricultural Research Station, Agricultural Research Center, Egypt (31°5′12″N, 30°56′49″E) during two growing seasons (2017–2018 and 2018–2019). The experiment was performed in a complete randomized block design with four replications to ensure homogeneous spread of the disease, where all entries were tested in every block, and inoculation of the spreader plants was performed. The sowing date was 28 November in each year. Seeds of the tested accessions were sown in 3‐m‐long rows, 30 cm apart, and at 5 g seed per row. The experiment was surrounded by a 1.5‐m^2^ belt of the highly susceptible universal variety ‘Morocco’ that served as a spreader of infection to promote homogeneous spread of the disease and provide a susceptible check. All cultural agronomic practices were applied as recommended in Egypt for the wheat crop. Surface irrigation (about four or five irrigations) and fertilization were applied. Superphosphate fertilizer was applied before sowing at the rate of 37 kg P_2_O_5_ ha^−1^, potassium fertilizer was applied before sowing at the rate of 37 kg K_2_O ha^–1^ in the form of potassium sulphate (48% K_2_O), and nitrogen fertilizer at the rate of 185 kg N ha^–1^ was applied in three doses in the form of urea (46.5% N); the first dose (one‐fifth of the dose) was added at sowing with irrigation, the second dose (two‐fifths of the dose) was added with the first irrigation (21 d after sowing), and the third dose (two‐fifths of the dose) was added with the second irrigation (25 d after the second dose). Pest and weed control were also applied as recommended. The yellow rust epidemic was initiated by artificial inoculation of spreader plants with urediniospores of *Pst* isolates at the growth stage BBCH‐37 (seven nodes detectable) (Lancashire et al., 1991) according to the method described by Tervet and Cassell (1951). The inoculum represented a mixture of the prevailing *Pst* races in Egypt during the respective season, including the aggressive race *PstS2*. A fine spray moisturized plants with water, which then were dusted with urediniospore powder mixture (one volume of fresh urediniospores and 20 volumes of talcum powder) at sunset before the onset of dew. Rust scoring was performed in spring 2018 and 2019 at the disease onset until the early dough stage (around BBCH‐83) when the highly susceptible check variety Morocco reached maximum rust severity. Adult plant reactions were noted as rust severity. Rust severity was noted as the percentage of coverage of all infected leaves with rust pustules (1, 5, 10, 20, 30, 40, 50, 60, 70, 80, 90 and 100%) following Cobb's scale as modified by Peterson et al. (1948).

### 2.5 Statistical processing of phenotypic data

In all experiments with phenotypic replications (two replications in Quedlinburg 2018; four replications for each year for field and seedling tests in Egypt), the arithmetic mean of all available replications for each trial was calculated and used for further statistical analysis, including calculation of the Pearson's product–moment correlations among different trials and years, as well as the QTL analysis. Additionally, best linear unbiased estimations were calculated, which were practically identical to the arithmetic means, indicating that no field effects were present.

### 2.6 Genotyping and QTL analysis

DNA was extracted from both populations’ seedlings according to the method of Doyle and Doyle (1990). Both DH populations were genotyped with a custom‐designed 15k Infinium array by the company TraitGenetics GmbH in Gatersleben, Germany, resulting in 3,567 polymorphic markers for DH Population 1 (103 genotypes) and 3,457 polymorphic markers for DH Population 2 (148 genotypes). The 15k Infinium array represents an optimized version of the 90k Infinium array (Muqaddasi et al., 2017; Soleimani et al., 2020; Wang et al., 2014). Data filtering and linkage map construction were performed as previously described (Muqaddasi et al., 2019). For quality control, SNPs harboring more than 20% missing values were removed and a $\chi^{2}$‐test (*P *< .001) was applied to remove SNPs deviating from the expected segregation pattern (i.e., 1:1). The genetic linkage map was constructed by selecting markers from each chromosome according to a consensus map (Wang et al., 2014). A linkage map was created in Join Map v4.0 (Van Ooijen, 2006) via maximum likelihood mapping with the logarithm of odds values ranging from 3.0 to 8.0.

The “Single Trait Linkage Analysis (Single Environment)” tool in the software package Genstat (Edition 19) was used to determine the QTLs separately for each trial by interval mapping without any cofactors. As a significance threshold, –log_10_(*P*‐value) > 3
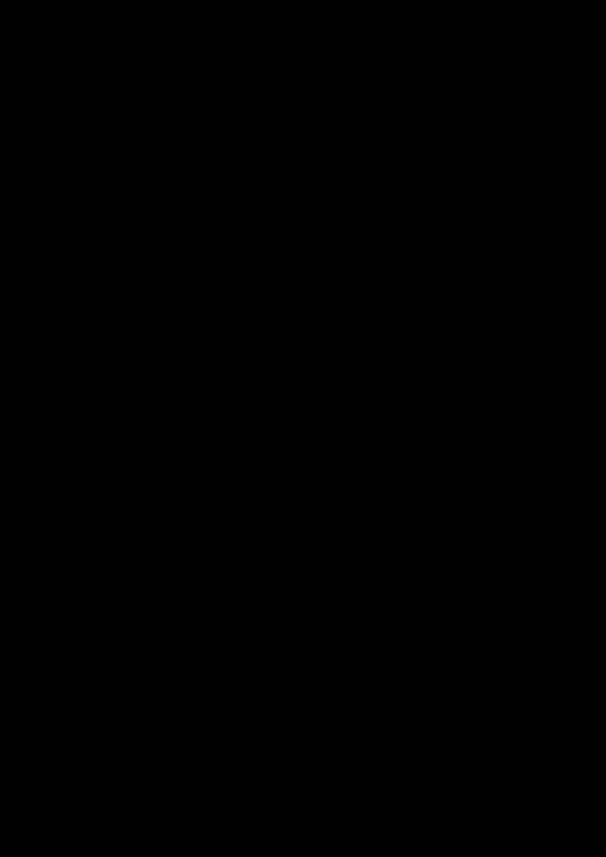
was abitrarily fixed. Only consistent QTLs which were detected in at least two environments were considered further. In addition, a second cutoff threshold based on Bonferroni correction for multiple testing was applied. This threshold was calculated by dividing *P* < .05 by the number of markers per population, and resulted in thresholds of –log_10_(*P*‐value) *> *4.85 for DH Population 1 and –log_10_(*P*‐value) > 4.83 for DH Population 2.

### 2.7 Connection of QTLs with the reference sequence and delimitation of QTL regions

The available sequences underlying the significantly linked markers were (Wang et al., 2014) and blasted against the wheat reference sequence v1.1 at the EnsemblPlants website (https://plants.ensembl.org/Triticum_aestivum/Info/Index) to identify the corresponding genes. To determine the physical target regions of the major QTLs, the SNP markers with the highest significance scores were used for blasting; the physical regions delimited by these markers were considered. The software package Geneious Prime (https://www.geneious.com/prime/) was used to extract the gene content of the respective genomic regions from published resources of the International Wheat Genome Sequencing Consortium (https://www.wheatgenome.org/).

## 3 RESULTS

### 3.1 Description of the phenotypic data

**FIGURE 1:**
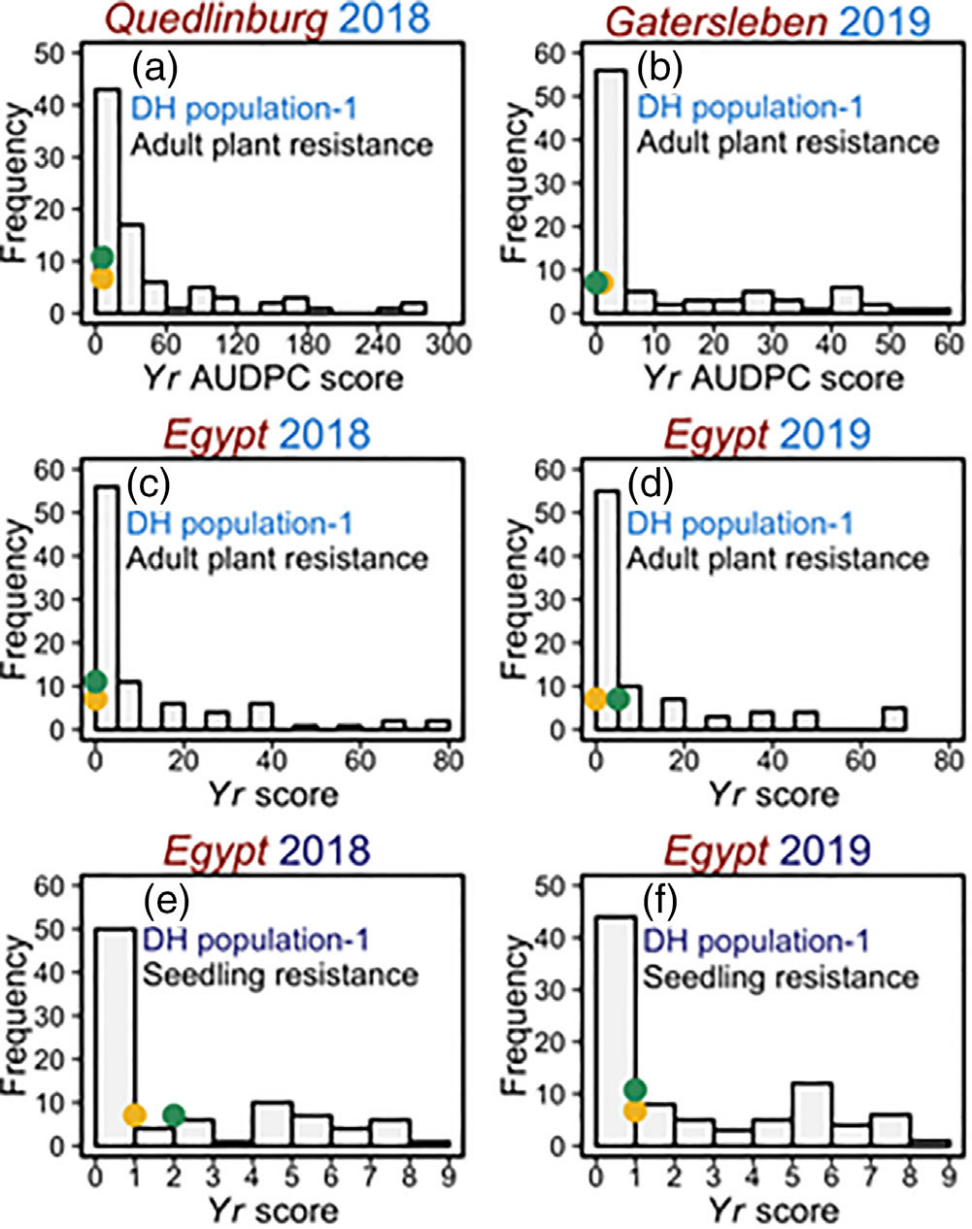
Phenotypic distribution of yellow rust infection in doubled haploid (DH) Population 1 in four field environments and two seedling tests. Note that different scales for yellow rust resistance (*Yr*) score were used. Orange and green dots within the figure represent the parental lines TRI‐11082 and TRI‐5645, respectively. (a) Quedlinburg 2018; (b) Gatersleben 2019; (c) Egypt 2018 adult plant resistance; (d) Egypt 2019 adult plant resistance; (e) Egypt 2018 seedling resistance; (f) Egypt 2019 seedling resistance

**FIGURE 2:**
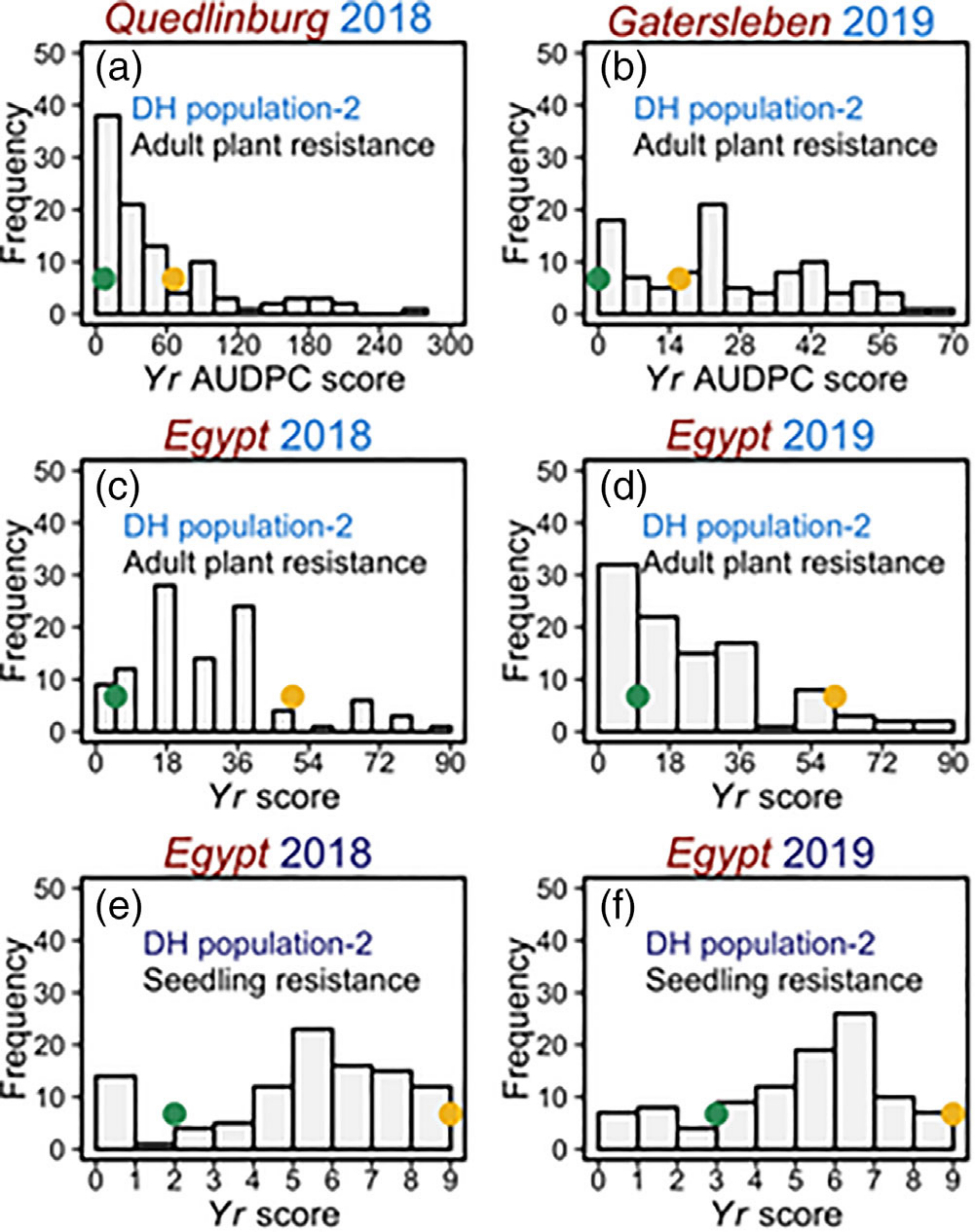
Phenotypic distribution of yellow rust infection in doubled haploid (DH) Population 2 in four field environments and two seedling tests. Note that different scales for yellow rust resistance (*Yr*) score were used. Orange and green dots within the figure represent the parental lines TRI‐10703 and TRI‐5310, respectively. (a) Quedlinburg 2018; (b) Gatersleben 2019; (c) Egypt 2018 adult plant resistance; (d) Egypt 2019 adult plant resistance; (e) Egypt 2018 seedling resistance; (f) Egypt 2019 seedling resistance

The assessment of resistance to yellow rust in two DH spring wheat populations was based on two field trials in Germany, two field trials for APR in Egypt, and two tests for seedling resistance in Egypt. In all cases, the phenotypic distributions in DH Populations 1 and 2 were quantitative but failed the test for normality at *P* < .001 (Figure 1 and Figure 2). A bimodal phenotypic distribution is indicative of the presence of major genetic factors. The parental lines of DH Population 1 (i.e., TRI‐11082 and TRI‐5645) were resistant in all tests in Egypt and in all field tests in Germany; however, the fact that segregation for resistance was observed in the DH progeny lines indicated that different resistance loci were present in both parental lines. For DH Population 2, the parental line TRI‐5310 was resistant in all tests in Egypt and field tests in Germany, whereas the parental line TRI‐10703 was susceptible in all tests.

**FIGURE 3:**
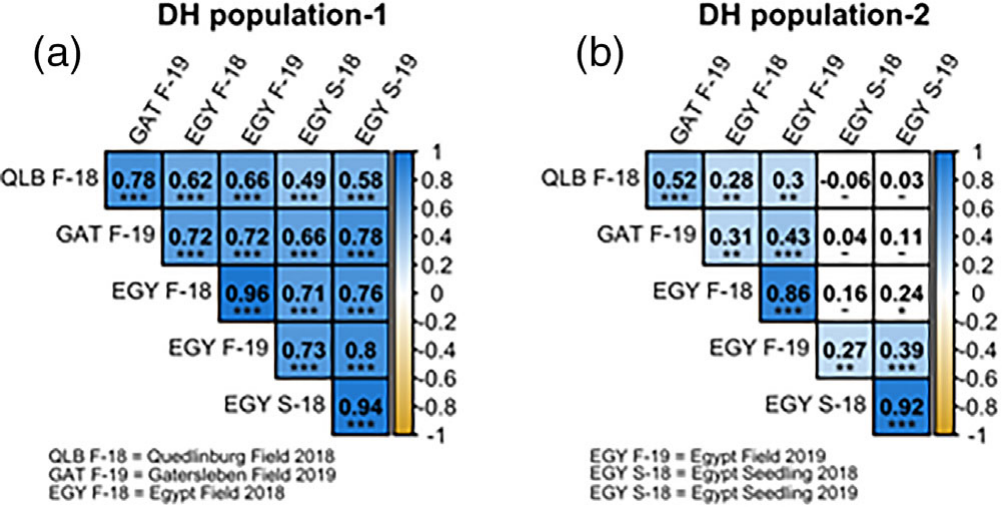
Pearson's product moment correlation of yellow rust resistance (*Yr*) scores for all field and seedling tests for (a) doubled haploid (DH) Population 1, and (b) DH Population 2. Asterisks denote the significance of the correlations: *P < .05, **P < .01, and ***P < .001; – denotes the absence of a significant correlation.

For DH Population 1, high correlations were observed among all field and seedling tests, ranging from *r* = 0.96 to 0.49 (Figure 3). In DH Population 2, high correlations were only observed between both field tests in Germany (*r* = 0.51), between both field tests in Egypt (*r* = 0.86), and between the two seedling tests (*r* = 0.93), whereas correlations among the German and Egyptian field tests were low with no significant correlations between the field and seedling tests.

Calculation of the repeatability values of replications within one environment resulted in values of 0.97 to 1.0, indicating the robustness of the phenotypic data. The pairwise heritabilities for seedling and field tests over 2 yr in Egypt amounted to 0.97 and 0.98 for DH Population 1, and 0.96 and 0.92 for DH Population‐2, respectively. The environments in Germany and Egypt represented different pathotype spectra; therefore, no best linear unbiased estimations or best linear unbiased predictions across environments were calculated but we considered each trial separately, compared the results, and considered only the consistent QTL detected in at least two environments.

### 3.2 Analysis of QTLs for resistance to yellow rust

**FIGURE 4:**
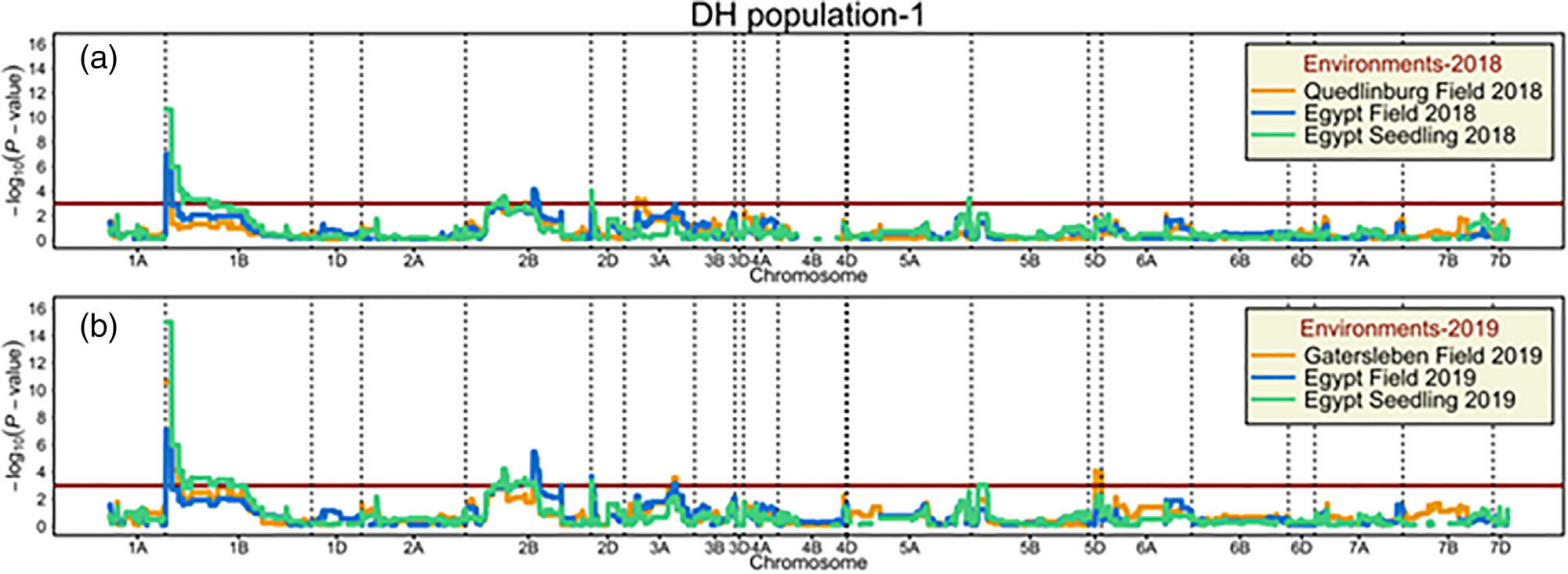
Quantitative trait locus (QTL) map of yellow rust resistance for doubled haploid (DH) Population 1. (a) Yellow rust QTL map for three environments in the year 2018; (b) yellow rust QTL map for three environments in the year 2019. The red horizontal line marks the significance threshold [–log_10_(*P*‐value) > 3] to detect the QTLs. Color coding is given in the figure legends

The QTL analysis was conducted on 3,567 and 3,457 SNP markers in DH Population 1 and DH Population 2. A highly significant QTL on chromosome 1B was detected in all field and seedling trials for DH Population‐1 (Figure 4). This QTL explained up to 28% of the phenotypic variation (*R* ^2^) in the field tests and up to 39% in the seedling tests; in both cases, the resistance source was TRI‐5645. All significant markers with –log_10_(*P*‐value) > 3 are listed in Supplemental Table S1, and Table 1 provides a summary of all QTL locations, which we detected at least twice. To ensure an even more stringent threshold, we included a second cutoff, which was a Bonferroni correction for multiple testing (*P* < 0.05) and which resulted in a –log_10_(*P*‐value) > 4.85 for DH Population 1 and –log_10_(*P*‐value) > 4.83 for DH Population 2. All QTLs passing the Bonferroni threshold are marked with asterisk in Table 1 and included all environments for the major QTLs on chromosme 1B and one environment for a QTL on chromosome 2B. Additional minor QTLs not passing the Bonferroni threshold were detected in DH Population 1 on chromosomes 2B, 2D, and 3A. A resistance QTL on chromosome 2B derived from TRI‐11082 explained up to 9% of the phenotypic variation in field resistance and 12% for seedling resistance.

**TABLE 1:** Consistent quantitative trait loci (QTLs) detected for resistance against yellow rust in two doubled haploid (DH) spring wheat populations. Position (Pos.) indicates the maximum of the consistent QTL in cM

				**Environment** ^b^							
**DH population**	**QTL**	**Chr**.^a^	**Pos. (cM)**	**QLBF‐18**	**GATF‐19**	**EGYF‐18**	**EGYF‐19**	**EGYS‐18**	**EGYS‐19**	**% *R* ^2^** ^c^	**Resistance source**
1	*QYr.ipk‐1B*	1B	0	4.9*	10.6*	7*	7*	10.6*	15*	28^d^; 39^e^	TRI 5645
1	*QYr.ipk‐2B.1*	2B	87	NS	3.4	3	3.3	3.6	4.2	9^d^; 12^e^	TRI 11082
1	*QYr.ipk‐2B.2*	2B	115	NS	NS	4.1	5.5*	NS	3.2	NA	TRI 11082
1	*QYr.ipk‐2D*	2D	0	NS	NS	NS	3.7	4	3.4	NA	TRI 5645
1	*QYr.ipk‐3A*	3A	123	NS	3.6	NS	3.1	NS	NS	NA	TRI 11082
2	*QYr.ipk‐6B*	6B	49	7.3*	13.2*	NS	3.4	NS	NS	36^α^	TRI 5310

* Significance of the corresponding QTL as–log_10_ (*P*‐value) > 4.85 (DH Population 1) or > 4.83 (DH Population 2).

^a^ Chr., chromosome; NS = nonsignificant; NA = data not available.

^b^ Environments: QLB F‐18, Quedlinburg field 2018; GAT F‐19, Gatersleben field 2019; EGY F‐18, Egypt field 2018; EGY F‐19, Egypt field 2019; EGY S‐18, Egypt seedling 2018; EGY S‐19, Egypt seedling 2019.

^c^ *R* ^2^ = percentage of phenotypic variation imparted by the corresponding QTL.

^d^ Phenotypic variation under field conditions.

^e^ Phenotypic variation at seedling stage.

**FIGURE 5:**
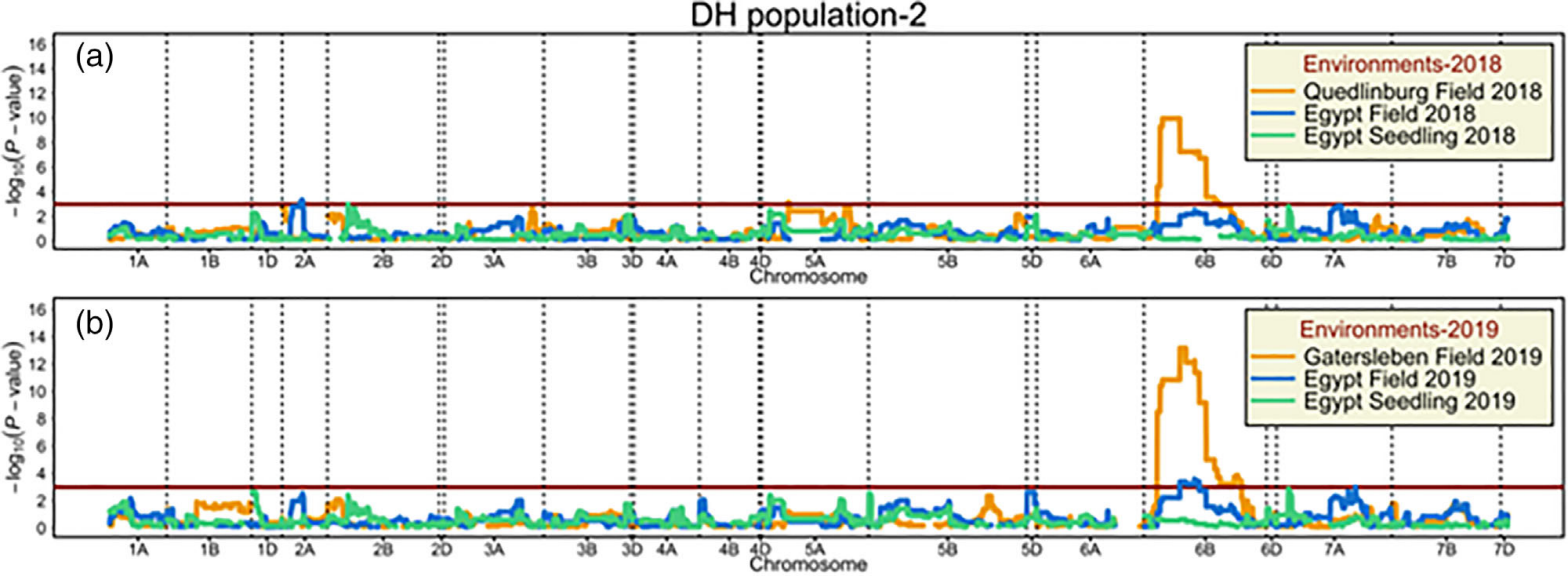
Quantitative trait loci (QTL) map of yellow rust resistance for doubled haploid (DH) Population 2. (a) Yellow rust QTL map for three environments in the year 2018, (b) yellow rust QTL map for three environments in the year 2019. The red horizontal line marks the significance threshold [–log_10_(*P*‐value) > 3] to detect the QTLs. Color coding is given in the figure legends

In DH Population 2, a major QTL above the Bonferroni threshold on chromosome 6B was detected in the two German field trials explaining up to 36% of the phenotypic variation (Figure 5). The same QTL was detected in one of the Egyptian field trials with –log_10_(*P*‐value) = 3.4, but no seedling resistance was detected. The resistance source was TRI‐5310 (Table 1).

**FIGURE 6:**
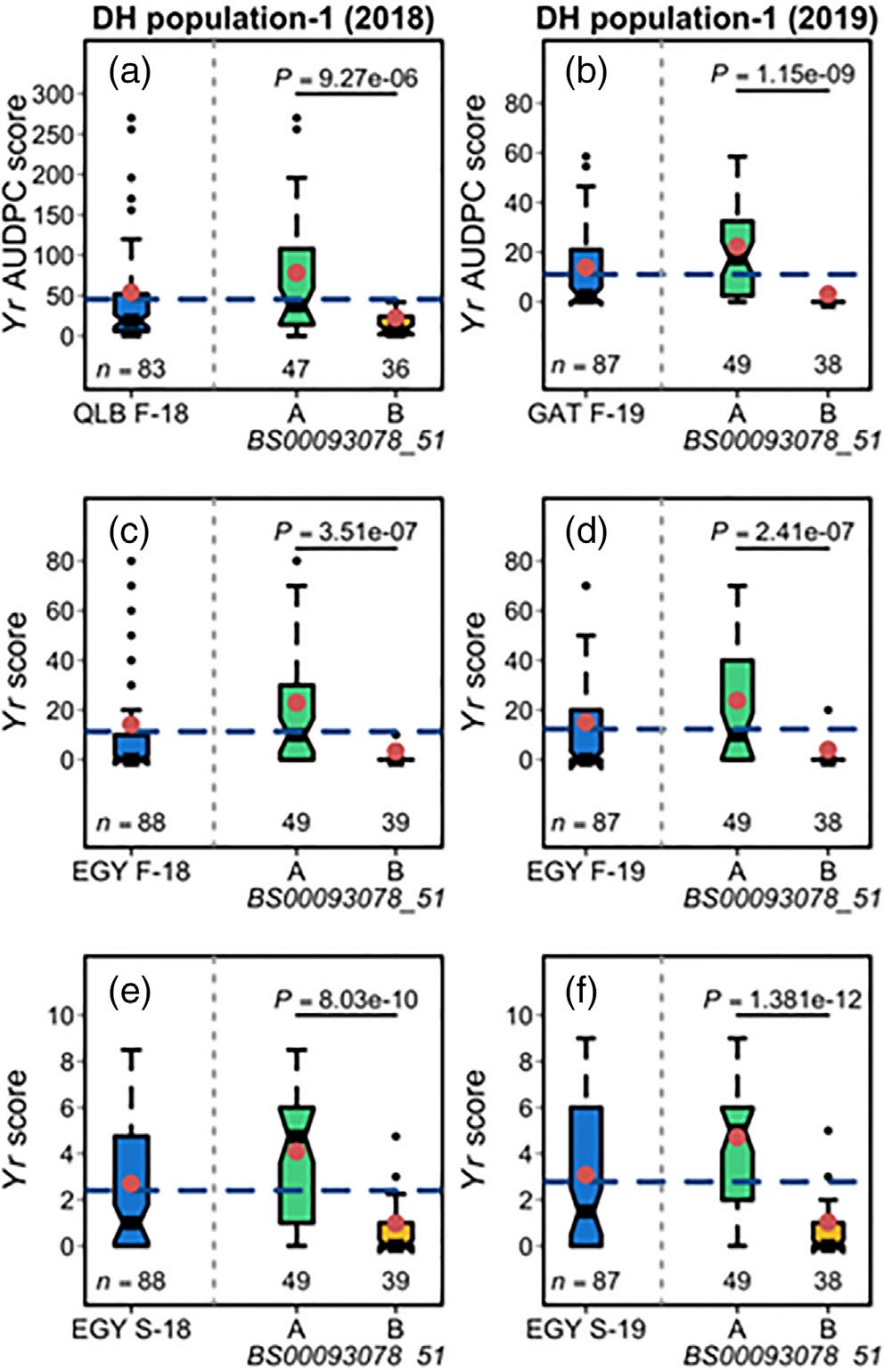
Allelewise phenotypic distribution of yellow rust resistance (*Yr*) scores in doubled haploid (DH) Population 1 based on the significant marker *BS00093078_51* on chromosome 1B. A represents the lines carrying the alleles of the parental line TRI‐11082, B represents those of the parental line TRI‐5645, the first boxplots represent the distribution of the full set of genotypes, the *P*‐value denotes the significance value of the two‐sided *t*‐test used to compare the mean values of the marker alleles, and red dots within the boxplots represent the mean of the respective population. (a) Quedlinburg 2018; (b) Gatersleben 2019; (c) Egypt 2018 adult plant resistance; (d) Egypt 2019 adult plant resistance; (e) Egypt 2018 seedling resistance; (f) Egypt 2019 seedling resistance

**FIGURE 7:**
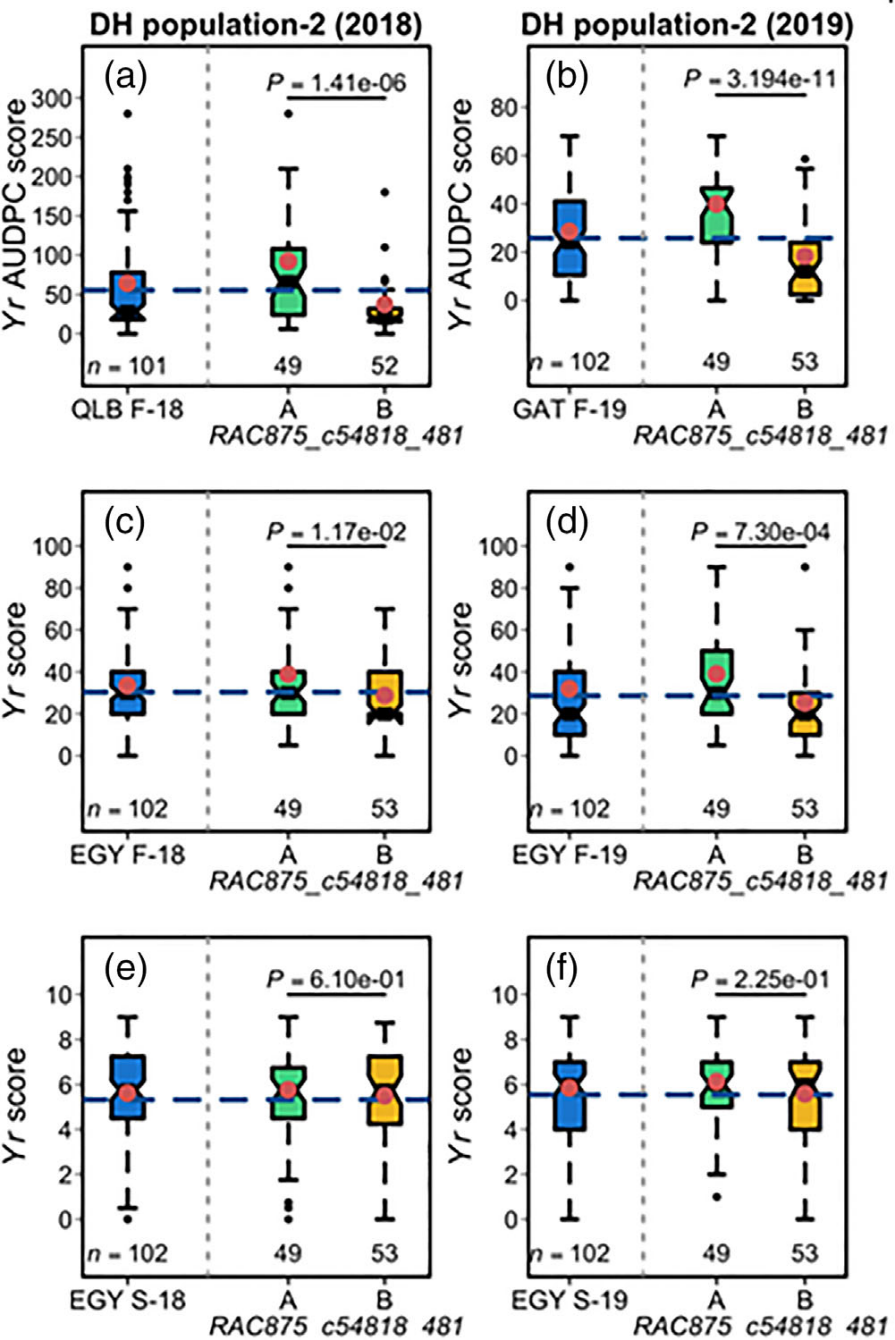
Allelewise phenotypic distribution of yellow rust resistance (*Yr*) scores in doubled haploid (DH) Population 2 based on the significant marker *RAC875_c5418_481* on chromosome 6B. A represents the lines carrying the alleles of the parental line TRI‐10703, B represents those of the parental line TRI‐5310, the first boxplots represent the distribution of the full set of genotypes, the *P*‐value denotes the significance value of the two‐sided *t*‐test used to compare the mean values of the marker alleles, and red dots within the boxplots represent the mean of the respective population. (a) Quedlinburg 2018; (b) Gatersleben 2019; (c) Egypt 2018 adult plant resistance; (d) Egypt 2019 adult plant resistance; (e) Egypt 2018 seedling resistance; (f) Egypt 2019 seedling resistance

The results of the QTL analyses were supported by the allelewise comparison in the form of whisker‐and‐box plots. Figure 6 shows the allele‐wise phenotypic distribution of the yellow rust score based on the highly significant marker BS00093078_51 on chromosome 1B in DH Population 1. In all trials, the lines carrying the TRI‐11082 allele showed a wide distribution, and most lines in this category were highly susceptible lines, whereas the lines carrying the TRI‐5645 allele were mainly in the resistant range. The comparison of the phenotypic means was significant (*P* < .001) in all cases, according to Welch's two‐sided *t*‐test. For DH Population 2, the same analyses were performed for the highly significant marker RAC875_c54818_481 on chromosome 6B (Figure 7). Significant differences (*P* < .001) between the two distributions were only observed in the two German field trials and the field trial 2019 in Egypt.

### 3.3 Anchoring of the major QTLs onto the physical map of wheat

The sequences of the most significant markers of the two major QTL on chromosome 1B for DH Population 1 and chromosome 6B of DH Population 2 were blasted onto the reference genome of wheat to identify the corresponding genes (Table 2). The most significant region for DH Population 1 ranged from 1,203,582 to 4,352,718 bp on chromosome 1B, with a clustering of markers between 1,203,582 and 1,784,048 bp. This interval contained 18 annotated genes from *TraesCS1B01G000800* to *TraesCS1B01G002400* (Supplemental Table S1). The most significant region of DH Population 2 extended on chromosome 6B from 144,887,799 to 149,418,405 bp; this interval harbored 35 annotated genes from *TraesCS6B01G144800* to *TraesCS6B01G148200*.

**TABLE 2:** Anchoring of the most significant markers onto the physical map of wheat

**Doubled haploid population**	**Marker name**	**Gene identifier**	**Chromosome**	**Physical location bp**	**Gene annotation**
1	*BS00093078_51*	*TraesCS1B02G000800*	1B	1,203,582–1,207,214	Peroxisomal membrane protein PEX14
1	*Excalibur_c10657_796*	*TraesCS1B02G001000*	1B	1,248,713–1,255,880	Tripartite motif‐containing protein 29
1	*Excalibur_c12376_569*	*TraesCS1B02G001000*	1B	1,248,713–1,255,880	Tripartite motif‐containing protein 29
1	*Kukri_c26168_423*	*TraesCS1B02G001500*	1B	1,416,127–1,418,511	Receptor‐like kinase
1	*Excalibur_c21898_1423*	*TraesCS1B02G001600*	1B	1,419,308–1,422,174	Adenylyl cyclase‐associated protein 1
1	*BS00050522_51*	*TraesCS1B02G002400*	1B	1,778,773–1,784,048	disease resistance protein (TIR‐NBS‐LRR class)
1	*BS00076192_51*	*TraesCS1B02G002400*	1B	1,778,773–1,784,048	disease resistance protein (TIR‐NBS‐LRR class)
1	*BS00022504_51*	*TraesCS1B02G008000*	1B	4,345,220–4,352,718	Mei2‐like protein
1	*BS00022505_51*	*TraesCS1B02G008000*	1B	4,345,220–4,352,718	Mei2‐like protein
1	*BS00083177_51*	*TraesCS1B02G008000*	1B	4,345,220–4,352,718	Mei2‐like protein
1	*Kukri_c38553_173*	No gene	NA^a^	NA	NA
1	*Kukri_c8390_1102*	No gene	NA	NA	NA
1	*Excalibur_c29707_318*	no gene	NA	NA	NA
1	*RAC875_c400_1363*	no gene	NA	NA	NA
1	*RAC875_c400_193*	no gene	NA	NA	NA
2	*RFL_Contig5844_291*	*TraesCS6B02G144800*	6B	144,887,799–144,893,244	DNA‐binding protein BIN4
2	*RAC875_c54818_481*	*TraesCS6B02G145900*	6B	146,138,556–146,145,479	Pathogenesis‐related homeodomain protein
2	*Kukri_c43406_2491*	*TraesCS6B02G146600*	6B	147,577,483–147,581,652	Nucleoporin
2	*Ra_c14171_2765*	*TraesCS6B02G146600*	6B	147,577,483–147,581,652	Nucleoporin
2	*wsnp_Ex_c19467_28423331*	*TraesCS6B02G146600*	6B	147,577,483–147,581,652	Nucleoporin
2	*wsnp_Ku_c5891_10414090*	*TraesCS6B02G146600*	6B	147,577,483–147,581,652	Nucleoporin
2	*wsnp_Ku_rep_c69252_68560829*	*TraesCS6B02G146600*	6B	147,577,483–147,581,652	Nucleoporin
2	*RAC875_c14171_664*	No gene	6B	147,582,221–147,582,331	NA
2	*wsnp_Ra_c50264_54965028*	*TraesCS6B02G146700*	6B	147,582,352–147,584,239	Structural maintenance of chromosomes protein 2‐1
2	*BobWhite_c18550_159*	No gene	6B	147,585,019–147,585,129	NA
2	*Kukri_c30417_305*	*TraesCS6B02G146800*	6B	147,677,525–147,681,024	GATA transcription factor
2	*Kukri_c14191_351*	No gene	6B	148,828,634–148,828,744	NA
2	*RAC875_c79742_264*	*TraesCS6B02G148100*	6B	149,057,547–149,060,279	Exocyst complex component EXO70A1
2	*RAC875_c4254_364*	*TraesCS6B02G148200*	6B	149,415,305–149,418,405	Protein phosphatase 2C
2	*wsnp_Ex_c64847_63484965*	*TraesCS6B02G148200*	6B	149,415,305–149,418,405	Protein phosphatase 2C

^a^ NA, not applicable.

## 4 DISCUSSION

### 4.1 Phenotypic distribution and linkage mapping results indicate the presence of major genetic factors for yellow rust resistance

Resistance to many fungal pathogens, including yellow rust, can be conferred by major resistance genes or multiple genetic factors with minor effects, usually termed QTLs. Although the segregation of a major resistance gene results in a bimodal distribution (Börner et al., 2000), a normal distribution is typical for a quantitative genetic inheritance pattern. Therefore, the phenotypic distribution in the progeny of a biparental cross indicates the mode of inheritance of a trait. Besides the normal and bimodal distributions, mixed types represent a bimodal but skewed distribution; these usually result from the segregation of a few major genes or a major gene modified by some minor factors (Haile et al., 2012). In both of our populations, a mixed type of bimodal distribution in all environments indicated major genetic factors, namely, major resistance genes segregating in the populations. These observations were confirmed by the results of QTL mapping in biparental populations–a method that is suitable for detecting minor effects and the segregation of major genes. Both parental lines of DH Population 1 had a resistant phenotype, but the presence of segregation in the progeny lines indicated that different resistance loci were present in both lines. This was confirmed by QTL mapping, where various major and minor QTLs for resistance were contributed by both parental lines (Table 1).

### 4.2 Mapping populations derived from the genetic resources were suitable for the discovery of major QTLs for yellow rust resistance

Genetic resources stored in the genebanks worldwide have been considered a valuable source for adding novel alleles into the existing elite germplasm genepool (Tanksley & McCouch, 1997). Multiple studies reported the evaluation of wheat germplasm for resistance to yellow rust (Badoni et al., 2017; Bulli et al., 2016; Elbasyoni et al., 2019; Huerta‐Espino et al., 2020; Juliana et al., 2020; Maccaferri et al., 2015; Tahir et al., 2020). Our study identified two major QTLs in the DH mapping populations derived from the spring wheat accessions of the federal ex situ genebank of the Leibniz Institute of Plant Genetics and Crop Plant Research Gatersleben in Germany. In DH Population 1, accession TRI‐5645, a landrace from Iran, was the source of a favorable allele for a major resistance QTL on chromosome 1B in the field as well as in the seedling tests. The field resistance was detected in (a) two field tests in Germany, where spray inoculation with the Warrior race (*PstS10*) had been conducted, and (b) in two field tests in Egypt where a mixture of the prevailing *Pst* races (including the aggressive *PstS2*) was used for inoculation (Table 1). The other parent of DH Population 1, namely TRI‐11082, a variety from the former German Democratic Republic, contributed minor resistance QTLs on chromosomes 2B and 3A (Table 1). In DH Population 2, a major QTL on chromosome 6B was contributed by the line TRI‐5310, representing the variety Eureke from France. This QTL was mainly effective in the German environments, although no resistance QTL was observed in the seedling tests and only a moderate resistance QTL in the field tests in Egypt. A possible reason for this could be the different geographic regions for the dominant *Pst* races. For example, Hovmøller (2019) showed that the Warrior race (*PstS10*) dominates in many European countries, whereas *PstS1* and *PstS2* dominate the rust populations in African countries.

### 4.3 Specific resistance responses reflect the diversity of pathotypes

The phenotypic trait of virulence is more important than any other trait for obligate biotrophic fungi such as *Pst* because they cannot survive on the primary crop host without virulence to the resistance genes in the cultivars (Liu et al., 2017). Avirulence–virulence patterns (Simmonds & Rajaram, 1988) were reflected by the host response and have guided wheat breeding programs aiming to design which resistance genes to target in specific wheat‐growing areas. In our study, the diversity of *Pst* pathotypes prevalent in the two study countries influenced the specific resistance responses. In Egypt, the high diversity of *Pst* pathotypes reported the predominant races 64E0 (virulent against *Yr4* and *YrSu*), 0E16 (virulent against *Yr8* and *Yr19*), 66E0 (virulent against *Yr4*, *Yr7*, *Yr22*, *Yr23*, and *YrSu*), 4E130 (virulent against *Yr2*, Yr6, *Yr7*, *Yr25*, and *YrHVII*), 2E0 (virulent against *Yr7*, *Yr22*, and *Yr23*), 2E16 (virulent against *Yr7*, *Yr8*, *Yr19*, *Yr22*, and *Yr23*), 4E0 (virulent against *Yr2 and Yr6*), 6E4 (virulent against *Yr2*, *Yr6*, *Yr7*, *Yr22*, *Yr23*, and *Yr25*), and 70E4 (virulent against *Yr2*, *Yr4*, *Yr6*, *Yr7*, *Yr22*, *Yr23*, *Yr25*, and *YrSu*); no virulence was detected against *Yr1*, *Yr5*, and *Yr10* (Draz, 2019b). The common lineage of the contemporary *Pst* population in Egypt belongs to the predominant aggressive race *PstS2* (virulent against *Yr2, Yr6–Yr9, Yr25*, and *Yr27*) that was used in the present study, but no virulence spectra were found for the Warrior race (virulent against *Yr1*–*Yr4*, *Yr6*, *Yr7*, *Yr9*, *Yr17*, *Yr25*, *Yr32*, and *YrSp* (Draz, 2019a, 2019b; Draz et al., 2018).

The aggressive, high temperature‐adapted race *PstS2* and the new virulent races *PstS10* and *PstS7* [Warrior (–), Warrior] are the most widely spread pathotypes of *Pst* covering geographical regions from Asia to Northern Europe (Hovmøller et al., 2016; Tadesse et al., 2014; Tehseen et al., 2021). The Warrior race was first discovered in the UK in 2011 and is currently the most prevalent race of *Pst* in Europe (Ali et al., 2017). The Warrior race has dominated the *Pst* population in Germany since 2015, and a few resistance genes (*Yr5*, *Yr8*, *Yr10*, *Yr15*, *Yr24*) are still effective (Losert et al., 2017). The resistance specificity is controlled by major race‐specific genes, categorized as seedling (all‐stage) resistance or race‐specific APR (Chen, 2013). In our study, the high level of specific resistance responses to *Pst* pathotypes based on the chromosome 1B QTL may be attributed to the major resistance gene *Yr10*, as outlined in the next paragraph. Both differential sets included this resistance, and virulence against *Yr10*, was rarely observed on the respective isolates and could not be analyzed (Hovmøller, 2019). Wheat lines exhibiting seedling and APR against both the *PstS2* race in Egypt and the Warrior race in Germany possess the wide‐spectrum resistance to yellow rust and should be utilized in breeding programs.

### 4.4 Exploiting the reference sequence of wheat led to the identification of candidate genes

Numerous QTLs and genes for yellow rust resistance have been published. However, most studies have been based on different reference populations and marker systems, which hampers a meaningful comparison. An early review of QTLs in wheat was provided by Rosewarne et al. (2013), based on simple sequence repeat markers. They reported three QTL regions on chromosome 6B. However, a direct comparison with our results is difficult because of different marker systems. The genomewide association study of Maccaferri et al. (2015) in hexaploid spring wheat reported a QTL on chromosome 6B at 60.1 cM on the basis of the marker *IWA2090*. According to our BLAST results, this marker corresponded to the gene *TraesCS6B02G189800* located at 221.4 Mb on chromosome 6B, whereas our QTL is located at 6B (144.9–149.4 Mb) (Table 2). The same article indicated the resistance genes *Yr35* and *Yr36* on chromosome 6B, and *Yr9* and *Yr10* on chromosome 1BS. A very early report mentioned the resistance gene *Yr10* located on chromosome 1B derived from an Iranian landrace (Kema, 1992). Moreover, in our study, an Iranian landrace was the source for the major QTL on chromosome 1BS.

The availability of a widely used set of mostly gene‐derived SNP markers (Wang et al., 2014) has revolutionized genetic mapping and genomewide association studies in wheat. The possibility of anchoring the sequences of significant markers to the wheat reference genome (The International Wheat Genome Sequencing Consortium et al., 2018) by blasting the most significant markers of the genetic map allows us to check the gene content of the respective genomic regions. It also enables a much more precise comparison with other published genes and QTLs, provided they used the same set of markers.

For the major QTL on chromosome 1B, we identified a genomic region containing ∼18 annotated genes in the interval between 1,203,582  and 1,784,048 bp as the physical region containing a cluster of the most significant SNP markers (Table 2 and Supplemental Table S1). We cannot completely exclude the possibility that our target gene lies outside this region, but it is currently the best assumption with the available marker density. The two genes *TraesCS1B01G002300* and *TraesCS1B01G002400* in this genomic region were both annotated as disease resistance protein (Toll‐interleukin‐like receptor–nucleotide binding site–leucine‐rich repeat class) and therefore represented potential candidates for the disease resistance gene (Supplemental Table S1). However, the five genes *TraesCS1B01G000100* to *TraesCS1B01G000500* were also annotated as disease resistance genes. They are located at the tip of chromosome arm 1BS and are close to the most distal significant marker *BS00093078_51* (Supplemental Table S1). The resistance gene *Yr10* derived from wheat line ‘PI 178383’ and the wheat variety ‘Moro’ was mapped at the tip of chromosome arm 1BS (Wang et al., 2002). Later, a unique and evolutionary conserved coiled‐coil domain–nucleotide binding site–leucine‐rich repeat sequence was identified as encoding the *Yr10* gene (Liu et al., 2014). We blasted the published sequence (AF149112.1) against the reference genome and identified the gene *TraesCS1B01G000200* as a BLAST hit in this genomic region (Supplemental Table S1). Some doubts about the functionality of the *Yr10* gene sequence were recently published, and a fine mapping approach relocated the gene in the proximity of the SNP marker *BS00050522_51* corresponding to *TraesCS1B01G002400* (Yuan et al., 2018). Genetic mapping in this genomic region has been hampered by an inversion in some wheat lines. Another broad‐spectrum resistance gene on chromosome 1BS is *Yr15*, derived from wild emmer wheat [*Triticum dicoccoides* (Körn. ex Asch. & Graebn.) Schweinf.], which encodes a putative kinase–pseudokinase protein, designated as wheat tandem kinase 1 (Klymiuk et al., 2018). However, *Yr15* is located more proximally at 25 to 93 Mb of chromosome Ta1BS and is therefore not considered as a potential candidate gene for the observed QTL. As a conclusion, *Yr10* is a likely candidate gene for the observed QTL on chromosome 1BS.

For the second major QTL in DH Population 2, the genomic region from 144,887,799 to 149,418,405 bp on chromosome 6B was identified as harboring the most significant SNP markers. The gene *TraesCS6B01G148400*, close to the significant marker *RAC875_c4254_364* (corresponding to *TraesCS6B01G148200*), was annotated as a disease resistance protein (nucleotide binding site–leucine‐rich repeat class) family and therefore is a potential candidate gene. On chromosome 6B, the resistance gene *Yr36* has been located and identified as a kinase‐START gene (*WKS1*) (Fu et al., 2009). Its slow rusting resistance phenotype was originally detected at relatively high temperatures (25–35 °C); however, partial resistance was also obtained at temperate conditions in the UK (Segovia et al., 2014). By blasting the published *WKS1* sequence (KT834963.1) onto the reference genome, we hit the genes *TraesCS6B02G010400* and *TraesCS6B02G010600* at 6.2 Mb, which are both far from our genomic region of interest; however, the blasting was relatively unspecific and identified numerous loci on other chromosomes. In Uauy et al. (2005), *Yr36* was genetically mapped onto an introgressed chromosome segment derived from *T. dicoccoides*, which may not be present in the reference sequence. Therefore, we tested the primer sequences of the microsatellite markers *Xgwm193* and *Xgwm508*, flanking this introgression (Uauy et al., 2005). They defined a region at 457 to 477 Mb on chromosome 6B which does not overlap with our region of interest. Therefore, we concluded that the major QTL on chromosome 6B does not represent *Yr36*. Numerous QTLs for *Yr* resistance were previously mapped on chromosome 6B [summarized in (Bulli et al. (2016)]; however, because of the different reference populations and marker systems, direct comparisons are difficult.

Recently the molecular identification of a cluster of three *Yr* genes (*Yr5*, *Yr7*, and *YrSP*) was reported on the long arm of chromosome 2BL at 682 to 688 Mb (Marchal et al., 2018). Our consistent minor QTL at 87 cM on chromosome 2B (Table 1 and Supplemental Table S1) had a physical location at 167 Mb and is therefore not connected to the reported cluster of *Yr* genes. Maccaferri et al. (2015) listed two resistance genes, *Yr55* and *Yr54*, on the long arm of chromosome 2D, whereas our minor QTL was located at 0 cM on the short arm of chromosome 2D (Table 1 and Supplemental Table S1). No major *Yr* genes but only QTL were reported for chromosome 3A (Maccaferri et al., 2015).

These examples show that the availability of sequence‐based high‐density SNP chips and the wheat reference sequence speed up identification of the physical regions underlying QTLs. Specific resistance genes are often introgressed from specific wheat accessions or even wild species and may not be represented in the reference sequence. For further functional validation and molecular characterization of resistance genes, sequencing of the source variety is required.

## 5 CONCLUSION

Testing for field and seedling resistance to yellow rust in two DH spring wheat populations identified two major QTLs on chromosomes 1B and 6B. Both QTLs were effective for the *PstS10* race Warrior in German field trials, and the QTL on 1B was also effective against the Egyptian pathotype spectrum and in seedling tests. The chromosome 1B QTL may represent *Yr10*, although for the chromosome 6B QTL, no known *Yr* gene was identified. This research demonstrated the usefulness of genetic germplasm resources as mapping populations in connection with modern genomic resources such as high‐density SNP chips and wheat reference sequences to identify novel resistance genes that are useful for breeding.

## AUTHOR CONTRIBUTIONS

Ibrahim S. Draz: Investigation; Methodology; Writing‐original draft; Writing‐review & editing. Albrecht Serfling: Investigation; Methodology; Writing‐review & editing. Quddoos H. Muqaddasi: Formal analysis; Methodology; Software; Validation; Writing‐review & editing; Conceptualization.

## CONFLICT OF INTEREST

The authors declare no conflict of interest

## Supporting information

Supplemental Table S1. List of significant markers [–log_10_(*P*‐value) > 3.0] for single environments and gene content of the major QTL based on the reference sequence (*XLXS*).
